# P-379. Timing Variations for Surgical Prophylaxis Within One Hour Pre-Incision: Impact on Surgical Site Infections in Elective Colorectal Surgery Procedures

**DOI:** 10.1093/ofid/ofae631.580

**Published:** 2025-01-29

**Authors:** Curtis D Collins, Eric Hartsfield, Robert K Cleary, Michael Veve, Kara K Brockhaus

**Affiliations:** Trinity Health Ann Arbor, Dexter, Michigan; Michigan Medicine, Ann Arbor, Michigan; Trinity Health Ann Arbor, Dexter, Michigan; Henry Ford Health, Detroit, Michigan; Trinity Health Ann Arbor, Dexter, Michigan

## Abstract

**Background:**

The optimal timing of surgical infection prophylaxis (SIP) is generally recommended to be given 60 minutes, or in some cases 120 minutes, prior to incision. Conflicting results have hampered further optimization of prophylaxis timing within this timing window. This study aimed to assess the impact of alternative prophylaxis timing on surgical outcomes, particularly surgical site infections (SSIs).

**Methods:**

A large, validated database of elective colorectal surgeries in Michigan was used to perform a multi-center, retrospective cohort study. Cohorts included patients who received SIP within 0-15 minutes and 16-60 minutes prior to incision. Adult patients who received colorectal surgeries spanning from July 2012 to June 2021 were included. Emergent or urgent surgeries, missing follow-up and antibiotic information, and patients who did not receive all prophylaxis prior to incision were excluded. The primary outcome was incidence of SSIs. A multivariable logistic regression model analyzed the impact of timing and other confounding variables on SSIs.

**Results:**

A total of 21,061 procedures were included. Of these, 6,405 patients (30.4%) received prophylaxis 0-15 minutes pre-incision and 14,656 (69.6%) received prophylaxis within 16-60 minutes. Cohorts were generally similar with differences in patients receiving β-lactam prophylaxis (97.1% vs 96%; *P* = < 0.001), mechanical and oral bowel preparation (64.5% vs. 67; *P* = 0.023), and adherence to recommended dosing (82% vs. 83.7%; *P* =0.002). There was no difference between cohorts in total SSIs (6.8% vs. 7.1%, *P* = 0.419) or CDI (1.1% vs. 1%; *P* =0.324); however, deep SSIs were increased in the 16–60-minute cohort (0.8% vs. 1.1%; *P* = 0.032). Multivariable logistic regression showed no association between prophylaxis timing and SSIs (adjusted odds ratio (aOR) 0.98; 95% CI 0.86-1.13; *P* = 0.817).

**Conclusion:**

Timing differences (0-15 minutes vs. 16-60 minutes pre-incision) did not significantly impacts overall SSIs incidence in elective colorectal surgeries.

**Disclosures:**

**Robert K. Cleary, MD, FASCRS, FACS**, Intuitive Surgical, Inc: Honoraria

